# Plant use of the Maasai of Sekenani Valley, Maasai Mara, Kenya

**DOI:** 10.1186/1746-4269-2-22

**Published:** 2006-05-05

**Authors:** Rainer W Bussmann, Genevieve G Gilbreath, John Solio, Manja Lutura, Rumpac Lutuluo, Kimaren Kunguru, Nick Wood, Simon G Mathenge

**Affiliations:** 1 University of Hawaii, Lyon Arboretum, 3860 Manoa Rd., Honolulu, HI 96822, USA; 2 Arogya Inc., 508 El Paso St., Austin, TX 78704, USA; 3 Sekenani Camp, P.O. Box 15010-00509 Langata, Nairobi, Kenya; 4 Sekenani Camp, P.O. Box 15010-00509 Langata, Nairobi, Kenya; 5 University of Nairobi, Botany Department, P.O. Box 30197, Nairobi, Kenya

## Abstract

Traditional plant use is of tremendous importance in many societies, including most rural African communities. This knowledge is however, rapidly dwindling due to changes towards a more Western lifestyle, and the influence of modern tourism.

In case of the Sekenani Maasai, the recent change from a nomadic to a more sedentary lifestyle has not, thus far lead to a dramatic loss of traditional plant knowledge, when compared to other Maasai communities. However, in Sekenani, plants are used much less frequently for manufacturing tools, and for veterinary purposes, than in more remote areas. While the knowledge is still present, overgrazing and over-exploitation of plant resources have already led to a decline of the plant material available.

This paper examines the plant use of the Maasai in the Sekenani Valley, North of the Masaai Mara National Reserve. The Maasai pastoralists of Kenya and Tanzania use a large part of the plants in their environment for many uses in daily life. The plant use and knowledge of the Sekenani Maasai is of particular interest, as their clan, the "Il-Purko", was moved from Central Kenya to this region by the British Colonial Administration in 1904.

The results of this study indicate that despite their relocation 100 years ago, the local population has an extensive knowledge of the plants in their surroundings, and they ascribe uses to a large percentage of the plants found. One-hundred-fifty-five plant species were collected, identified and their Maa names and traditional uses recorded. Although fifty-one species were reported as of "no use", only eighteen of these had no Maasai name. Thirty-three were recognized by a distinctive Maa name. Thirty-nine species had a medicinal use, and 30 species served as fodder for livestock. Six species could not be identified. Of these plants five were addressed by the Maasai with distinct names. This exemplifies the Sekenani Maasai's in-depth knowledge of the plant resources.

Traditionally, the Maasai attribute most illnesses to the effect of pollutants that block or inhibit digestion. These pollutants can include "polluted" food, contact with sick people and witchcraft. In most cases the treatment of illness involves herbal purgatives to cleanse the patient. There are alsofrequent indications of plant use for common problems like wounds, parasites, body aches and burns.

## Background

Plants have been an integral part of life in many indigenous communities, and African communities are no exception. Apart from providing building materials, fodder, weapons and other commodities, plants are especially important as traditional medicines [1]. Many tribes in Africa have a sophisticated plant knowledge [2]. Western influences have led to an accelerating decline of this tradition [3]. Most knowledge is still transferred entirely orally in many communities. Despite the "Witchcraft Act" of 1925, outlawing traditional medicine in Kenya, the practice continued in secret, until parts of the law were revoked with independence in 1963 [1]. Western style healthcare supplied by the government has expanded in the last decades, but is still often not readily available, and many regions remain completely underserved. Consequently, most communities still use herbal remedies as a readily and cheaply available alternative.

The Maasai are originally nomadic pastoralists. "Maasai" indicates a speaker of the Maa language, which belongs to the Chari-Nile branch of the Nilo-Saharan language family. Maa speaking peoples migrated into their current territory around the 16^th ^century [4]. The Maasai are divided into 11 sections, each occupying specific areas. These sections show differences in dialect and ceremonial life [5]. At the end of the 19^th ^century their original grazing territory encompassed almost 80 percent of Kenya and Northern Tanzania. During colonial rule, much of this land was lost to agriculture. Some sections of the Maasai were even moved completely from their homelands.

By agreement with his Majesty's Commissioner for the East African Protectorate in 1904/1911, the Il-Purko Maasai were moved South to the area around Narok [5,6]. The Maasai in the Sekenani area belong to this group. The Il-Purko, like other sections of the tribe, traditionally form homesteads, called Manyattas or Engkangs. These settlements consist of a ring of low huts surrounded by a thorn- or wooden fence. Manyattas are used for 4–5 years, and are then abandoned [7]. Originally nomadic, the life of the Maasai has undergone profound changes in the last decades. Now, many Maasai live a sedentary lifestyle, and the formerly communally owned land is being subdivided into group ranches or family units. This change in settlement pattern, together with rising populations and a continuously stronger outside influence, has had a profound impact on the Maasai's lifestyle and environment [8,9]. Maasai life is still very interwoven with the natural surroundings. This is illustrated by the intricate knowledge about the surrounding environment.

Tourism has become a major economic force in Kenya in the last decades. The Maasai Mara in particular has seen a tremendous increase in visitors and provides major income for the country, but the distribution of this wealth to the local communities is scarce [10].

The main pillars of Maasai diet are still milk and blood from cows, and soups derived from wild collected herbs. Berries and other wild fruits supplement the diet. Both are eaten mostly by women and children. Herbal knowledge is widespread in the community. Families are often able to care for their own health. Traditional healers "laibon" are mostly responsible for the treatment of "witchcraft", and have an important ceremonial function [5].

Most Bantu speaking peoples in East Africa believe that illness is related to a curse from deceased ancestors. In contrast, the Maasai attribute most illnesses to the effect of pollutants that block or inhibit digestion. These pollutants can include "polluted" food, contact with sick people and witchcraft. In most cases, the treatment of illness involves herbal purgatives to cleanse the patient. There are also frequent indications of plant use for common problems like wounds, parasites, body aches and burns.

Mayor health concerns for the Sekenani Maasai include malaria, gastro-internal disorders, parasites, tuberculosis, brucellosis and Sexually Transmitted Diseased (STD's). Skin problems, burns, wounds and fractures are lesser problems associated with the daily dangers of livestock keeping.

Early accounts of Maasai plant use date back to the beginning of the last century [11,12]. Studies on Maasai Ethnobotany of the Mara region have mainly focused on the forested areas of the Loita Hills [13,14]. The most detailed study on the plant use of a Maasai community was conducted in Loita in 2000 [14] and was used as main comparison for this study.

## Methodology

### Study area

**Figure 1:**
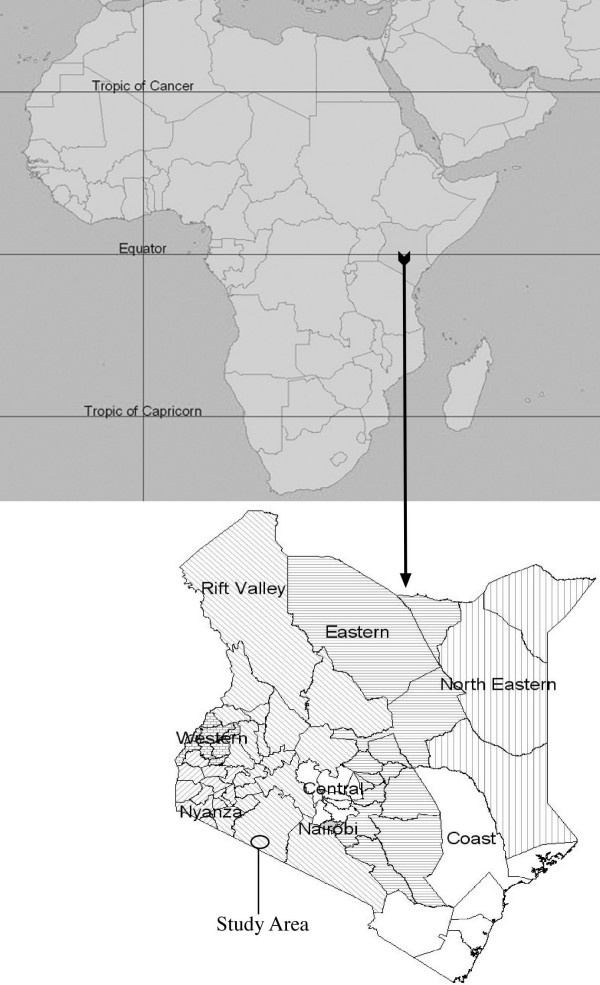
Map of Kenya and location of the study area.

The Sekenani Valley is located in the Northeastern corner of the wider Maasai Mara region, just outside the popular Mara Game Reserve (Fig. 1). Twenty-four mostly sedentary Maasai families occupy Sekenani.

The region receives about 600 mm annual rainfall with peaks in April and December.

The vegetation consists, to a large extent, of **Grassland**, with Poaceae forming the main vegetation layer, interspersed with few annuals and perennials, and occasional trees and shrubs, mostly *Acacia *sp. Theses grasslands derive from Evergreen Bushland under constant grazing and fire pressure. Soils are mainly black cotton soils. **Wooded Grassland **shows a very similar appearance, however bush cover increases up to 40 percent. In **Evergreen Bushland**, shrubby vegetation and tree islands cover more than 40 percent of the ground.

### Data collection

In 1994 the authors began fieldwork in the Sekenani area, and were thus well acquainted to the local population. The 24 Maasai families in the Sekenani valley are all stakeholders in Sekenani Safari Camp, the main base for all study activities. After years of field visits to the area, the local community asked the authors to conduct an inventory of the ethnobotanical knowledge of the valley. The main collection of ethnobotanical data occurred 2002.

Many ethnobotanical studies use questionnaires to interview segments of the population about their ethnobotanical knowledge. Frequently interviewees are asked to name plants they know, and to reveal the use of the respective species. Sometimes informants are accompanied to the field to collect plant material. This methodology easily misses plants found at further distances from the villages. It often includes only a part of the population, and it gives no indication about which percentage of the flora is actually used.

To avoid these shortfalls, and to obtain a more detailed inventory of plant use, the authors tried to collect as many plant species as possible. After collecting the material the plants were photographed and pressed in Sekenani Camp. Ethnobotanical data was collected by interviews with the four Maasai co-authors (John Solio, Manja Lutura, Rumpac Lutuluo, Kimaren Kunguru). Interviews were conducted directly in the field, during collection trips, and by examination of the freshly collected specimens with members of the 24 families of Sekenani. Interviewees were chosen without distinction of gender, and after seeking oral consent from each respondent.

During the interviews, a standardized set of questions was used to inquire about each plant the authors collected. Showing interviewees the collected plants and asking them questions about each plant was preferred over asking them to recall, from memory, which plants they used. This method was preferred over pure questionnaires because it enabled information gathering about species that are known by the community, but no longer used. The authors believe that it was important to gather this information about these "unknown" ore "useless" species to document that knowledge has already been lost, and to preserve the knowledge of traditional names. All interviews were carried out with at least one of the local co-authors as interpreter and assistant.

All plant species encountered were collected, and dried and processed at the University of Nairobi Herbarium. The specimens are numbered in the collection series "RBUGGG", and the collection numbers are given in [Additional file 1].

RW Bussmann and S. Mathenge identified the plant samples, and all voucher specimens are deposited at the University of Nairobi Herbarium. The nomenclature of all plants follows: for herbs [15], for trees, shrubs and lianas [16], for Cyperaceae [17], and [18] for all families.

Data on plant species, families, vernacular names, parts used, traditional use and modality of use were recorded and are compared in Appendix 1 to plant uses reported by Maundu et al. [14] for the Loita community.

## Results and discussion

### Traditional nomenclature

Like in many traditional societies, Maasai plant nomenclature is complex. Plant names are mostly related to plant appearance and use.

It is very common that one vernacular name refers to multiple species. "*Olosida*" for example, refers to the majority of Acanthaceae, "*Enkaiieiedyia" *to all Commelinaceae, and *"Ososian*" to Pteridophytes. Maasai plant nomenclature is much more intricate than the nomenclature of many other communities. Many plants are referred to with a specific name, especially if a plant has a specific use. In addition, names might indicate morphological characteristics or habitat. Since Maa is originally not a written language, there is no agreed upon correct spelling to the names. The spelling used in this paper represents a consensus of the Maasai co-authors and [19].

### Plant use

A total of 155 plant species belonging to 52 families were collected in the Sekenani Valley (Table 1). Of these, 149 could be identified (Additional file 1). This represents a large percentage of the total flora of the Sekenani Valley. All species encountered belong to the Grassland-Evergreen Bushland ecosystem.

**Table 1:** Plant families with number of species recorded in Sekenani and number of species with traditional use.

**Plant family**	**Species found**	**Species used**
Acanthaceae	11	2
Adianthaceae	2	1
Aizoaceae	1	1
Amaranthaceae	4	1
Anacardiaceae	2	2
Anthericaceae	1	1
Apocynaceae	3	2
Asclepiadaceae	2	2
Asparagaceae	2	2
Asphodelaceae	1	1
Aspleniaceae	1	0
Asteraceae	12	4
Canellaceae	1	1
Capparidaceae	2	1
Combretaceae	1	1
Commelinaceae	3	3
Commiphoraceae	1	0
Convolvulaceae	4	1
Crassulaceae	2	2
Cucurbitaceae	1	0
Cyperaceae	11	11
Dryopteridaceae	3	2
Ebenaceae	1	1
Euphorbiaceae	7	5
Fabaceae	9	6
Hyacinthaceae	1	1
Hypoxidaceae	1	1
Lamiaceae	6	3
Liliaceae	1	1
Loranthaceae	2	2
Malvaceae	3	3
Meliaceaea	1	0
Menispermaceae	1	1
Mimosaceae	3	3
Moraceae	1	0
Ochnaceae	1	1
Olacaceae	1	1
Oleaceae	1	1
Plumbaginaceae	1	1
Poaceae	12	12
Polygonaceae	1	1
Rhamnaceae	1	0
Rubiaceae	4	3
Rutaceae	3	3
Santalaceae	1	1
Sapindaceae	3	1
Scrophulariaceae	3	2
Solanaceae	1	1
Sterculiaceae	1	1
Tiliaceae	4	4
Verbenaceae	1	1
Vitaceae	2	2
Unidentified (indet.)	6	5

In comparison, 267 species were collected in the Loita Hills [14]. The respective study concentrated on the forest and forest-bushland transition zone and the species found represent only about 60 percent of the plants used in the higher Loita region [14]. The grassland and bushland zones were under-represented in the Loita study. One hundred seventy-three of the species in the Loita Hills study are mostly found in *Olea-Juniperus *forest and dense bushland; they do not occur in the Sekenani region. Sekenani and Loita potentially share only 94 species found in the Loita study [14]. Forty-three species were found in both studies. However, the Loita study [14] concentrated only on useful species, rather than the complete flora. An overview on the uses attributed to plants in Sekenani is given in Table 2.

**Table 2:** Use categories of plants used by the Sekenani Maasai, and number of individual plant species used for each category.

**Plant uses**	**Number of species used**
**No use**	51
**Medicinal**	39
**Fodder**	30
**Browsed by wild animals**	22
**Ceremonial**	21
**Construction**	17
**Food**	14
**Arms**	8
**Firewood**	7
**Tools**	6
**Veterinary**	5
**Bees**	4

### No use

Fifty-one species found in the Sekenani area had "no use" for the local Maasai community. Eighteen of these species had also no name in Maa. The most frequent families with useless species were Acanthaceae, Amaranthaceae and many Asteraceae; families that contain a high number of herbal species. This coincides with observations that traditional plant use for Maa speaking peoples focuses on woody species (for medicines, construction and firewood), and grasses (for fodder) [3].

### Medicinal species

Thirty-nine (25%) of the plant species encountered in Sekenani had some medicinal use (Table 3). This is lower than the 33%of species reported as used medicinally in Loita [14].

**Table 3:** Traditional medicinal uses of plants registered in Sekenani. Tabulated overview on use categories and number of species used for every medicinal category.

**Medicinal use category**	**Number of species used**
Dental hygiene (toothbrush)	12
Malaria	7
Strength (children)	4
Wounds	4
Hygiene (smell)	3
Soup (strength in adults)	3
Stomach problems	3
Tea and local beer	3
Skin diseases	2
Common cold	2
Chest problems	2
Joint and muscle pain	2
Parasites	2
"All diseases"	2
Venereal disease	1
Circulation	1
Depression	1

The most common medicinal uses in Sekenani included: Dental hygiene, malaria, and general strength and wound care. Like in more traditional Maasai communities [3], the plants used to cure diseases served mainly as strong purgatives and emetics; they "cleanse" the body and digestive system from polluting substances.

Although the number of plants used medicinally in Sekenani made up only 25 percent of all plants, this category was still statistically most important amongst all plant use types. Easy access to governmental health care (close to one of the main Mara Ranger Headquarters) and the hospital facilities in the provincial capital Narok, has led to a pronounced decline of plants used in disease treatment. "Everyday" uses, e.g. the use of fragrant sticks as efficient toothbrush have been better maintained.

### Dental hygiene

Almost a third of the plant species used medicinally in Sekenani were employed solely as toothbrush. The species used were mostly fragrant tree and shrub species (*Euclea divinorum, Croton dichogamus, Phyllanthus sepialis, Indigofera brevicalyx, I. swaziensis, Tephrosia hildebrandtii, Olea europaea, Grewia similes, G. tembensis*).

### Malaria

Although malaria treatment is often available at health centers, the traditional use of herbs for the treatment of "malaria and fever" is still common. The cures mostly involve the ingestion of purgative plant extracts, obtained by boiling plant material. In the Sekenani valleythe most important species used to treat malaria were *Achyranthes aspera, Warburgia salutaris, Combretum molle, Olea europaea, Sporobolus stapfianus, Teclea nobilis, Toddalia asiatica *and *Cissus quinquangularis*.

### Other medicinal uses

Apart from dental hygiene and malaria treatment, only few plant species were employed for treatment of other health conditions. These include "**Strengthening of children**" (*Ozoroa insignis, Cyphostemma serpens*), "**Wounds**" (*Cynanchum altiscandens, Jasminum abyssinicum, J. fulminense, Solanum incanum*), "**Good smell**", where plants were burned and the fumes either inhaled or the patient sat exposed to the fumes (*Blepharis stuhlmannii, Rhus natalensis, Tarchonanthus camphorates*), preparation of strengthening "**Soup**" for adults, especially with *Tarchonanthus camphorates *and *Combretum molle*, care of for "**Stomach problems**" (*Ximena americana, Sporobulus stapfianus*), preparation of "**Traditional tea and beer **(*Croton dichogamus, Ochna ovata, Osyris lanceolata*), "**Skin diseases**" (*Osteospermum vaillantii*), "**Common cold**", which was often seen synonymous to fever and malaria, (*Toddalia asiatica, Cissus quinquangularis*), "**Chest problems/Pneumonia**" (*Rhus natalensis, Conyza *sp., *Warburgia salutaris*), "**Joint and muscle pain**" (*Carissa edulis, Craterostigma plantagineum*), "**Parasites"**, which were generally treated by using plant parasites like Mistletoes (*Odontella schimperi, Phragmanthera rufescens*), "**Circulation**" (*Combretum molle*) and "**Low spirit / depression**", which was treated with a Morning Glory (*Evolvulus alsinoides*).

### Venereal disease

Plants for the cure of venereal diseases such as Gonorrhea, Syphilis and others, were almost negligible in Sekenani. In contrast, plants used to treat STDs were of significant importance in Loita [14].

### Fodder

Fodder for domestic animals is of paramount importance in a livestock keeping society. Most plant species used for fodder were addressed by distinct names. It is not surprising that the use for fodder had the highest statistical significance (19%) after medicinal plant use.

A large number of sedges and grasses (most importantly *Cyperus amauropterus, C. cyperoides, C. obtusifolius, C. vestitus, Mariscus remotus, Rhynchospora elegans, Brachiaria brizantha, Eragrostis braunii, Harpanche schimperi, Hyparrhenia hirta, Loudetia kagerensis, Panicum maximum, Rhynchelytrum repens, Setaria plicatilis, Sporobolus festivus*, and *Urochloa insculpta*) were mentioned as preferred fodder plants. A large number of plant species with high water content were explicitly used for forage during the dry season (*Delosperma nakurense, Ipomoea tenuirostris, Crassula pentandra, Bulbostylis boeckleriana, Phyllanthus sepialis, Glycine wightii, Plectranthus longipes, Cissamphelos mucronata, Acacia hookeri, Polygonum salicifolium, Grewia bicolor, G. tembensis*).

### Browsed by wild animals

Species browsed by wild animals had no significance in the Loita study[14]. In striking contrast, almost as many species were named in this category in Sekenani as used for domestic animals. One reason for this distinction might be the fact that many Maasai in Sekenani are involved with game tourism, and thus wildlife may have a higher importance than in Loita.

### Ceremonial

Ceremonial plant use is of principal importance in daily Maasai life. Many species have a specific ceremonial significance, generally associated with blessings, age-rites and witchcraft. The most important ceremonial plant was Olive (*Olea europaea*), which was used in all ceremonies, and is thought to bring good luck. Ferns (*Doryopteris concolor, Athyrium *sp., and others) were used to bless women.

A wide range of species, most importantly *Asparagus africanus, A. falcatus, Commelina africana, C. benghalensis *and *Cyanotis foecunda, Cyperus cyperoides*, were used to bless cows.*C. involucratus, Mariscus remotus *and *Lippia javanica*, were used in circumcision ceremonies. *Hibiscus aponeurus, Helichrysum gerberaefolium *played a role in witchcraft.

### Construction

Plants are also of vital importance in traditional home construction. Seventeen species were used. In Sekenani, most plants in this category were used to tie the sticks together to frame traditional huts (*Cyperus distans, C. pinguis, Hibiscus calyphyllus, Pavonia patens, Oplismenus compositus, Dombeya burgessiae, Grewia bicolor, G. tembensis*). The Sekenani Maasai used mostly spiny species for fence construction (*Chaetacme microcarpa, Acacia polyacantha, Pyrostria phyllanthoidea*). *Hyparrhenia hirta *was used as thatch. The scarce timber was needed as firewood. In contrast, timber was most often used for construction of houses and fences in Loita, due to the closeness to forest [14].

### Food

Fourteen plants identified in Sekanani were categorized as food, most importantly *Carissa edulis, Cyphostemma serpens, Euclea divinorum, Erythrococca bongensis, Ximena americana, Vangueria infausta, Grewia bicolor *and *G. tembensis*. The term food was generally used to refer to ripe fruits and berries eaten by women and children.

### Weapons

Hardwoods used to produce weapons included *Acalypha volkensii, Tinnea aethiopica, Albizia gummifera, Olea europaea, Pyrostria phyllanthoidea, Tarenna graveolens *and *Teclea nobilis*. The Sekenani Maasai live a more sedentary life, where defense is much less important than in former times. Carrying spears, swords, clubs (rungus), and sticks, is still culturally important. The typical weapon carried indicates the position of men in life. Spears are normally only carried by warriors (moran), elders carry sticks. Bows and arrows are more commonly seen in young boys. Clubs are virtually carried by every male, from small herding boys to elders. Weapons also still serve an important role in protection from wild animals.

### Firewood

Firewood is one of the most important commodities in the region, and the Maasai go to great efforts to collect it. The main species used were either hardwoods, or woods selected for their pleasant smell (*Tarchonanthus camphoratus, Euclea divinorum, Tinnea aethiopica, Albizia gummifera, Tarenna graveolens*).

### Tools

The use of plants to make tools showed the greatest difference between the Loita and Sekenani Maasai. In the remote Loita region, a large number of plant species were used to make everyday tools. In Sekenani, it is easy to reach main roads and the provincial capital. Therefore Western tools had mostly replaced traditional plant-based tools, and only a few species were still used to make tools.

### Veterinary

For both the Sekenani and Loita Maasai, access to governmental veterinary care has vastly reduced the need of plant-based medicines for livestock.

### Bees

Honey has an important role in Maasai society^5^. Plant species attractive to bees were thus clearly singled out in Sekenani. Four species were explicitly mentioned as "bee pasture", i.e. very important for honey production (*Barleria grandicalyx, Combretum molle, Acacia polyacantha*, and an unidentified species).

## Conclusion

Sekenani represents a Grassland and Evergreen-Bushland habitat. The data about plant use in these ecosystems give a more complete picture about Maasai plant use by supplementing other studies in the region [13,14] that have mainly focused on the ethnobotanical use of forested areas.

Traditional plant use and knowledge thereof is still essential to the Maasai families living in the Sekenani valley. Judging from the decrease of plants used in traditional tools and in veterinary medicine, it seems that the proximity to main roads, the Maasai Mara National Park and the general exposure to Western influence plant use has declined.

With changes in lifestyle and associated decline of the use of plants, it is the author's fear that Maasai ethnobotanical knowledge might continue to decline. In comparison to older studies, part of the Maasai plant knowledge has already disappeared^12^. Studies from the turn of the century registered almost 500 plant species used by the Maasai^12^. Since this knowledge is still mostly taught orally, without written record, the loss of knowledge is accelerating.

It is the author's hope that there will soon be an illustrated identification guide for Maasai plant use, best produced in Maa and Kiswahili. The local Maasai are owners of this traditional knowledge, and it would be of great benefit for future generations to have access to this knowledge.

## Authors' contributions

All authors share th se contributions to the fieldwork of this manuscript. S Mathenge and RW Bussmann identified the plant material. RW Bussmann analyzed the data and RW Bussmann and G. Gilbreath prepared the manuscript.

## Declaration of competing interests

The author(s) declare that they have no competing interests.

## Supplementary Material

Additional File 1 Plants used by the Sekenani Maasai
