# Supplementary material for: Plant use of the Maasai of Sekenani Valley, Maasai Mara, Kenya
Source: J Ethnobiol Ethnomed. 2006 May 5;2:22. doi: 10.1186/1746-4269-2-22 (PMC1475560; doi:10.1186/1746-4269-2-22)
Supplement: Additional File 1 — Plants used by the Sekenani Maasai [file 1746-4269-2-22-s1.doc]

Additional file 1: Plants used by the Sekenani Maasai

| **Family / Scientific name** | **Indigenous name** | **Use and parts used**  **(in bold uses reported by Maundu et al. [14])** | **Coll.# RBUGGG** |
| --- | --- | --- | --- |
| **Acanthaceae** |  |  |  |
| *Barleria grandicalyx* Lindau | Enkokii | Bees suck flower | 137 |
| *Blepharis stuhlmannii* Lindau | Orkunetiia | Boil plant and wash very young babies for smooth skin; gets rid of babies smell, flower can be sucked for sweet liquid **Maundu et al.: same** | 136 |
| *Crossandra nilotica* Oliver | Olosida | No use | 51/163 |
| *Crossandra subacaulis* C.B.Clarke | unknown | No use | 50 |
| *Dyschoriste radicans* Kuntze | unknown | No use | 59 |
| *Hypoestes triflora* Roem. & Schult. | Olosida | No use | 47 |
| *Justicia exigua* S. Moore | Olorook Kileleg | No use | 22 |
| *Justicia flava* Vahl. | Olosida | No use | 96 |
| *Justicia sp.* | Olosida | No use | 8 |
| *Phaulopsis imbricata* Sweet | Olosida | No use | 99 |
| *Thunbergia alata* Sims | unknown | No use | 62 |
| **Adiantaceae** |  |  |  |
| *Doryopteris concolor* (Langsd. & Fisch.) Kuhn | Ososian | For women in ceremony, old men use it to bless women | 56 |
| *Pellea sp.* | Enchani Osoitok | No use | 28 |
| **Aizoaceae** |  |  |  |
| *Delosperma nakurense* (Engl.) A.G.J.Herre | Epurda Ontiare | Only used for sheep to eat during dry season | 112 |
| **Amaranthaceae** |  |  |  |
| *Achyranthes aspera* L. | Olekidogo | 1. For malaria - remove roots, smash with water, drink, vomit  **Maundu et al.: fodder, roots boiled against malaria and syphilis** | 46 |
| *Celosia anthelmetica* Aschers | Esonkoyo | No use | 15 |
| *Cyathula uncinulata* (Schrad.) Schinz. | Olairepirepi | No use but when pods are dry they can enter the eyes and cause damage | 77 |
| *Psilotrichum elliottii* Baker | unknown | No use | 9 |
| **Anacardiaceae** |  |  |  |
| *Ozoroa insignis* Delile | Olokunonoi | Boil bark and give young children to be healthy | 149 |
| *Rhus natalensis* Krauss | Ormisigiyoi | 1. Warriors burn and then put under clothes for good smell; 2. Baboons eat the fruits; 3. Maasai eat the fruit to prevent chest problems **Maundu et al.: fruits edible, bark decoction for children as tonic and for stomach problems; leaves used during circumcision; twigs as toothbrush; fuel-wood** | 29,115 |
| **Anthericaceae** |  |  |  |
| *Chlorophytum sparsiflorum* Baker | Olagugua | When ready use bulb to seal jerry can  **Maundu et al.: glue prepared from roots** | 53 |

| **Family / Scientific name** | **Indigenous name** | **Use and parts used**  **(in bold uses reported by Maundu et al.^14^)** | **Coll.# RBUGGG** |
| --- | --- | --- | --- |
| **Apocynaceae** |  |  |  |
| *Carissa edulis* (Forssk.) Vahl. | Enkamuriaki | 1. Eat fruit; 2. For venereal diseases boil root, slaughter a ram and mix meat with the root, boil 2 hrs and eat. 3. Same for back and joint problems: drink liquid from boiling  **Maundu et al.: edible fruit, latex as chewing gum, roots boiled for gonorrhea, pelvic pain, back ache, fodder** | 127 |
| *Cyphostemma serpens* (A. Rich.) Descoigns | Olorodo | Only used by young children, boiled root makes them healthy; however, fruits eaten by everybody  **Maundu et al.: No use** | 111 |
| *Landolphia buchananii* Stapf. | Enchaituryian | No use | 75 |
| **Asclepiadaceae** |  |  |  |
| *Cynanchum altiscandens* K. Schum. | Ormeko Orsage | Bite leaf and put on wound  **Maundu et al.: young girls wear as ornament, string for building** | 78 |
| *Sarcostemma viminale* (L.) R. Br. | Ololei | Poisonous, milk makes you blind when it gets into the eyes **Maundu et al.: roots in soup for elders, latex for eye disease** | 16 |
| **Asparagaceae** |  |  |  |
| *Asparagus africanus* Lam. | Embereepapa | Wash away spirit of the person who has passed away: put in water and wash yourself, use with olive to sacrifice (drought), then burn  **Maundu et al.: to clean circumcised boys, as sieve** | 131 |
| *Asparagus falcatus L.* | Orkiar Enkure | 1. Fruits are on root; 2. Has much water, collect up to 20l; 3. Water tank  **Maundu et al.: leaves applied to cuts** | 38,151 |
| **Asphodelaceae** |  |  |  |
| *Bulbine abyssinica* A. Rich. | Alakuuyoi | The long roots are eaten by Guinea fowls and Baboons | 144 |
| **Aspleniaceae** |  |  |  |
| *Asplenium aethiopicum* (N.L. Burm.) Becherer | Enchani Osoitok | No use | 61 |
| **Asteraceae** |  |  |  |
| *Ageratum conyzoides* L. | Esonkuyo Enkare | Near river, No use | 88 |
| *Bidens pilosa* L. | unknown | No use | 74 |
| *Bidens sp.* | unknown | No use  **Maundu et al.: leaves and roots for malaria** | 145 |
| *Conyza sp.* | Olobaai | 1. Put into milk or water to wash goat kids to prevent ticks and flies; 2. Roots: for chest problems, boil in water, drink, then vomit. This cleans the chest; 3. When walking long hold in hand. It is believed that then food will come and that the animals will not see you | 129 |
| *Conyza sumatrensis* (Retz.) E. Walker | Oleturot | No use | 89 |
| *Crassocephalum picridifolium* S. Moore | unknown | No use | 87 |

| **Family / Scientific name** | **Indigenous name** | **Use and parts used**  **(in bold uses reported by Maundu et al.^14^)** | **Coll.# RBUGGG** |
| --- | --- | --- | --- |
| *Gutenbergia cordifolia* Oliv. | Nanurdelo | No use, poisonous flower, if it gets into the eye one has to see a doctor **Maundu et al.: No use** | 143 |
| *Helichrysum gerberaefolium* Sch. Bip. ex Hochst. | Sakutae | Young boys remove root and chew, if spat on someone the person will feel sleepy | 161 |
| *indet.* | unknown | No use | 153 |
| *Osteospermum vaillantii* (Decne.) Norlindh | Eleleshwa Ekop | For skin disease - boil whole plant, then put pot under you and steam, when sweating wash yourself (use OLOMEEI in the same way) | 139,170 |
| *Tarchonanthus camphoratus* L. | Esentyio, Killeleshua | 1. Bed - home or bush, has a good smell and protects from bed bugs; 2. Underarms - for smell and if you are tired; 3. Used leaves as soup; 4. Wipe sweat; 5. Good for lighting fire **Maundu et al.: inhalation of smoke for headache, dry leaves in water against tapeworm, women an children use leaves against sun, leaves in armpit as perfume, leaves used as towel, twigs for arrows, firewood, construction** | 142 |
| *Vernonia lasiopus* O. Hoffm. | unknown | No use | 138 |
| **Canellaceae** |  |  |  |
| *Warburgia salutaris* (Bertil. f.) Chiov. | Osokonoi | Against malaria and stomach ache. Put bark in cold water for 5 minutes, filter and drink **Maundu et al.: ground bark as emetic, for malaria and pneumonia; bark with fat given to mothers for easy delivery; bark for medicine against cold, stomachache, respiratory disorders, fever; fruits edible; timber; NOT firewood** | 108 |
| **Capparidaceae** |  |  |  |
| *Capparis fascicularis* DC. | Enkaturdei | All parts poisonous  **Maundu et al.: root ground and mixed with buffalo horn against enemies, to bewitch others** | 13 |
| *Maerua triphylla* A. Rich. | Enkamoloki | No use **Maundu et al.: fodder** | 41 |
| **Combretaceae** |  |  |  |
| *Combretum molle* G. Don. | Ormaroroi | 1. Root good for making soup; 2. Slaughter bull and make soup. This prevents malaria and circulatory problems; 3. Flowers are good for bees  **Maundu et al.: bark decoction as beverage, roots for backache, pelvic pains, gonorrhea, firewood** | 126 |
| **Commelinaceae** |  |  |  |
| *Commelina africana* L. | Enkaiieieyia | Used to treat cows. Only used by special old men. These put magic and then place where cows pass | 4 |
| *Commelina benghalensis* L. | Enkaiieieyia | Used to treat cows. Only used by special old men. These put magic and then place where cows pass  **Maundu et al.: for coughs and colds, spathe liquid against female infertility, female cleansing, fodder** | 30,148 |
| **Family / Scientific name** | **Indigenous name** | **Use and parts used**  **(in bold uses reported by Maundu et al.^14^)** | **Coll.# RBUGGG** |
| *Cyanotis foecunda* DC. ex Hassk. | Enkaiieieyia | Used to treat cows. Only used by special old men. These put magic and then place where cows pass | 27 |
| **Commiphoraceae** |  |  |  |
| *Boscia angustifolia* A. Rich | Oloireroi | No use **Maundu et al.: same** | 110 |
| **Convolvulaceae** |  |  |  |
| *Ecolvulus alsinoides* Willd. | unknown | No use | 171 |
| *Ipomoea cairica* (L.) Sweet | Olosida | No use | 97 |
| *Ipomoea tenuirostris* Choisy | Olopitaq / Olopito | 1. Eaten by goats and sheep when flower is in fruit; 2. For young Maasai girls to use for tying together a hut | 70,94 |
| *Ipomoea wightii* (Wall.) Choisy | Enkaisurutiai | No use **Maundu et al.: fodder** | 7,52 |
| **Crassulaceae** |  |  |  |
| *Crassula pentandra* Schoenland | Ormairo Giro | Found in the plains, sheep eat this during dry season as it does not dry out, gives them water | 162 |
| *Kalanchoe lanceolata* Pers. | Ekidosi | Children play removing leaves and bark to make something like a syringe | 26 |
| **Cucurbitaceae** |  |  |  |
| *Zehneria scabra* Sond. | Enkasirarai | No use | 85 |
| **Cyperaceae** |  |  |  |
| *Bulbostylis boeckleriana* (Schweinf.) Beetle | Eseat | Eaten by cows, sheep and buffalo | 155,165 |
| *Cyperus amauropus* Steud. | Enkurba Seai | 1. Guinea fowl, Francolin, Vervet and Baboons eat roots; 2. Cows eat stems; 3. Children play with parts of root, parents teach | 164 |
| *Cyperus circumclusus* (C.B. Clarke) Schweinf. | Enkonyou | Eaten by cows, sheep and buffalo | 34 |
| *Cyperus cyperoides* (L.) Kuntze | Eseyiia | 1. Fodder for domestic animals; 2. Place on side before crossing water when being circumcised; 3. Shows that you don't drink water because you drink only milk and juice when circumcised | 35 |
| *Cyperus distans*  L.f. | Oseyiai | 1. Joining sticks; 2. Eaten by elephants | 80 |
| *Cyperus involucratus* Rottb. | Olaimutai | Ceremonial, age rites, men use it to bless special boys during circumcision | 104 |
| *Cyperus obtusifolius* | Enkonyou | Eaten by cows, sheep and buffalo | 32 |
| *Cyperus pinguis*  (C.B. Clarke) Mattf. & Kuek. | Oseyiai | 1. Joining sticks; 2. Eaten by elephants | 73 |
| *Cyperus vestitus* Hochst. ex Krauss | Enkonyou | Eaten by cows, sheep and buffalo | 33 |
| *Mariscus remotus* C.B.Clarke & C.B.Clarke | Eseyiia | 1. Fodder for domestic animals; 2. Place on side before crossing water when being circumcised; 3. Shows that you don't drink water because you drink only milk and juice when circumcised | 37 |
| *Rhynchospora elegans* Kük. | Olopitaq | 1. Eaten by goats and sheep when flower is in fruit; 2. For young Maasai girls to use for tying together a hut | 140 |
| **Dryopteridaceae** |  |  |  |
| *Athyrium sp.* | Ososian | For women in ceremony, old men use it to bless women | 68 |
| *Ceterach cordatum* (Thbg.) Desv. | unknown | No use | 60 |

| **Family / Scientific name** | **Indigenous name** | **Use and parts used**  **(in bold uses reported by Maundu et al.^14^)** | **Coll.# RBUGGG** |
| --- | --- | --- | --- |
| **Ebenaceae** |  |  |  |
| *Euclea divinorum* Hiern | Enkiyei | 1. put meat on top of leaves to keep moist for 1-2 days; 2. Toothbrush; 3. Eat sweet berries; 4. Firewood  **Maundu et al.: roots boiled against malaria, tanning of leather, fruits edible, anthelmintic** | 23 |
| **Euphorbiaceae** |  |  |  |
| *Acalypha volkensii* Pax | Esiaeiti/Esiati | Arrows | 44,91 |
| *Bridelia micrantha* Baill. | Odapashi Ebenek | No use | 100 |
| *Chaetacme microcarpa* Rendle | Ontirkish | Fencing | 39,98,105 |
| *Croton dichogamus* Pax | Olokidigai | 1. Toothbrush; 2. Leaves used for Maasai beer; 3. Root flavors meat and gives strength | 43 |
| *Erythrococca bongensis* Pax | Nkayakug | Young children eat the fruit and like it very much **Maundu et al.: walking sticks** | 135B |
| *Phyllanthus sepialis* Müll.Arg. | Esampu Keke | 1. Toothbrush; 2. Eaten by goats and impala | 24 |
| *Sapium ellipticum* Pax | Enchaituryian | No use | 82 |
| **Fabaceae** |  |  |  |
| *Desmodium salicifolium* Mart. ex Benth. | Odapashi Ebenek | No use | 90 |
| *Dolichos oliveri* Schweinf. | unknown | No use | 141 |
| *Glycine wightii* (Wight & Arn.) Verdc. | Olopito | 1. Eaten by goats and sheep when flower is in fruit; 2. For young Maasai girls to use for tying together a hut | 132 |
| *indet.* | Etuwala | Teas are used as rattle, don't eat! | 154 |
| *Indigofera brevicalyx* Baker f. | Enchanai Okiken | Toothbrush when it is big | 118 |
| *Indigofera swaziensis* Bolus | Emeim | Toothbrush**Maundu et al.: construction, fodder** | 116 |
| *Indigofera volkensii* Taub. | Enkameriruni | No use | 159 |
| *Tephrosia hildebrandtii V*atke | Enchanai Okiken | Toothbrush when it is big | 113 |
| *Tinnea aethiopica* Hook. f. | Nebae | Used to make arrows and fire sticks **Maundu et al.: leaves against eye infections, ground with buffalo horn** | 114 |
| **Hyacinthaceae** |  |  |  |
| *Bowiea kilimandscharica* Mildbr. | Erpisia Lonkonoi | Use as strainer to filter soup | 119 |
| **Hypoxidaceae** |  |  |  |
| *Hypoxis obtusa* Burch. | Enkamalasai | Tuber used by children to make toy calabashes **Maundu et al.: *Hypoxis angustifolia* Lam. Used for same** | 128 |
| **Lamiaceae** |  |  |  |
| *Aeollanthus stormsii* Gürke | Ormairo Giro | No use | 124 |
| *Fuerstia africana* T.C.E.Fr. | Oloitodor Enkai | 1. Children remove leaves and chew like tobacco; 2. Produces red color for decoration | 12 |
| *Leonotis nepetifolia* (L.) R. Br. | Orbibi | 1. Birds like to suck flower, bird has same name as plant **Maundu et al.: children suck nectar** | 157 |
| *Orthosiphon somalensis* Vatke | Ormitaa | No use | 21 |
| **Family / Scientific name** | **Indigenous name** | **Use and parts used**  **(in bold uses reported by Maundu et al.^14^)** | **Coll.# RBUGGG** |
| *Plectranthus longipes* Baker | Ormairo Giro | Found in the plains, sheep eat this during dry season as it does not dry out, gives them water | 83,121 |
| **Liliaceae** |  |  |  |
| *Trachyandra saltii* (Baker) Oberm. | Aikuo | The long roots are eaten by Guinea fowls and Baboons | 133 |
| **Loranthaceae** |  |  |  |
| *Odontella schimperi* Tiegh. | Ormegaru Keon | Against parasites in pregnant women, use leaf, burn, smash it up, then lick a little bit, this helps women to not breath so heavy | 5 |
| *Phragmanthera rufescens* (DC.) Balle | Ormegaru Keon | Against parasites in pregnant women, use leaf, burn, smash it up, then lick a little bit, this helps women to not breath so heavy | 19 |
| **Malvaceae** |  |  |  |
| *Hibiscus aponeurus* Sprague & Hutchinson | Enkarani | 1. Grows straight but can not be used as cow stick because cows will die; 2. Used as stick to curse | 117 |
| *Hibiscus calyphyllus* Cav. | Osukupai Naibor | 1. Joining sticks; 2. Eaten by elephants | 72 |
| *Pavonia patens* (Andr.) Chiov. | Osupukai | To join sticks **Maundu et al.: root against diarrhea** | 152 |
| **Meliaceae** |  |  |  |
| *Turraea mombassana* C. DC. | unknown | No use **Maundu et al.: toothbrush, against dysentery, roots emetic, construction** | 122 |
| **Menispermaceae** |  |  |  |
| *Cissampelos mucronata* A.Rich. | Olopitaq/Olopito | 1. Eaten by goats and sheep when flower is in fruit; 2. For young Maasai girls to use for tying together a hut | 49,92 |
| **Mimosaceae** |  |  |  |
| *Acacia hookeri* Meisn. | Enchardalani | 1. Elephants and goats eat plant; 2. Bark is used for constructing a home; 3. Eat bark to prevent stomach ache | 174 |
| *Acacia polyacantha* Willd. | Orkigiro | 1. Fencing the village because has good thorns; 2. Eaten by elephants and goats; 3. Bees use plant | 173 |
| *Albizia gummifera* (J.F. Gmel.) C.A. Sm. | Osupakupe | 1. Strong tree, makes strong stick for ceremonies and walking; 2. Spears; 3. Bark for fire **Maundu et al.: fodder, timber, NOT as firewood** | 2 |
| **Moraceae** |  |  |  |
| *Ficus sur* Forssk. | Odapashi Ebenek | No use | 102 |
| **Ochnaceae** |  |  |  |
| *Ochna ovata* F. Hoffm. | Olodo Ganayioi | 1. Use leaves and bark for tea; 2. Fruit eaten by baboons **Maundu et al.: construction** | 120 |
| **Olacaceae** |  |  |  |
| *Ximena americana* L. | Lamania | 1. Ripe fruit eaten; 2. Boil branch for the young to drink, this is good for digestion **Maundu et al.: fruit edible; roots in soup or tea for health** | 40 |
| **Oleaceae** |  |  |  |
| *Jasminum abyssinicum* DC. | Ormeko Orsage | Bite leaf and put on wound **Maundu et al.: No use** | 25 |
| **Family / Scientific name** | **Indigenous name** | **Use and parts used**  **(in bold uses reported by Maundu et al.^14^)** | **Coll.# RBUGGG** |
| *Jasminum fluminense* Vell. | Ormeko Orsage | Bite leaf and put on wound | 84,169 |
| *Olea europaea* ssp. a*fricana* (Mill.) P. Green | Olorien | 1. Toothbrush; 2. Ceremonies - age to elder rites, use to make sacrifice (big fire); 3. Making rungus; 4. Spear handles; 5. Calf: boil and give to drink, this kills worms; 6. Mix with SOKONOI (green hut) this cures malaria, drink half liter; 7. Boys circumcision - put on house as sign **Maundu et al.: outer bark medicine for stomach upset, bark against colds, pneumonia, anthelmintic; fuel-wood; cleaning calabashes; rungus; sticks; ceremonial plant** | 168 |
| **Plumbaginaceae** |  |  |  |
| *Plumbago zeylandica* L. | Orgeyiantus | 1. Girls use to decorate: take sheep urine and mix together, make small cuts around eyes and rub in; 2. Mash together and express to make bigger marks **Maundu et al.: No use** | 156 |
| **Poaceae** |  |  |  |
| *Brachiaria brizantha* Stapf | Ormaguitian | Fodder for domestic animals | 95 |
| *Eragrostis braunii* Schweinf. | Empiris | Eaten by domestic animals | 36 |
| *Harpachne schimperi* Hochst. | Orioaraan | Eaten by livestock, when dry spikes can get into animals eyes | 6 |
| *Hyparrhenia hirta* (L.) Stapf | Orpesi Orasha | Eaten by domestic animals, for roofing | 79 |
| *Loudetia kagerensis* (K.Schum.) C.E.Hubb. | Enkujit | Eaten by cows, sheep and buffalo | 146 |
| *Oplismenus compositus* (L.) P.Beauv. | Empalakai | To join sticks | 66 |
| *Panicum maximum* Hochst. ex A.Rich. | Olmrisi | Eaten by animals | 31 |
| *Rhynchelytrum repens* (Willd.) C.E.Hubb. | Enkonyou | Eaten by cows, sheep and buffalo | 165 |
| *Setaria plicatilis* Hack. ex Engl. |  | Fodder | 55 |
| *Sporobolus festivus* Hochst. ex A.Rich. | Enyoil | Eaten by cows, sheep and buffalo | 167 |
| *Sporobolus stapfianus* Gand. | Enkonyoyo | Against malaria and stomach ache. Put bark in cold water for 5 minutes, filter and drink | 109 |
| *Urochloa insculpta* Stapf | Enoporori | Fodder for domestic animals | 93 |
| **Polygonaceae** |  |  |  |
| *Polygonum salicifolium* Willd. | Enkujiria | Fodder for domestic animals | 86 |
| **Rhamnaceae** |  |  |  |
| *Helinus mystacinicus* (Ait.) Steud. | unknown | No use **Maundu et al.: root decoction mixed with animal fat for gonorrhea** | 67,76 |
| **Rubiaceae** |  |  |  |
| *Pentanisia ouranogyne* S.Moore | Elauo Ekop | No use | 150 |
| *Pyrostria phyllanthoidea* (Baill.) Bridson | Enkogo Olntim | 1. Use for fencing when a big tree; 2. Use for making rungus | 123 |
| *Vangueria infausta* Burch. | Orgomei | 1. Fruit eaten by baboons, monkeys, humans and especially children | 81 |
| **Rutaceae** |  |  |  |
| *Tarenna graveolens* (S. Moore) Brem. | Ormasei | 1. When dry good firewood; 2. Good for rungus **Maundu et al.: No use** | 3,45 |
| **Family / Scientific name** | **Indigenous name** | **Use and parts used**  **(in bold uses reported by Maundu et al.^14^)** | **Coll.# RBUGGG** |
| *Teclea nobilis* Del. | Ogrilai | Toothbrush, making rungus **Maundu et al.: sticks** | 106 |
| *Toddalia asiatica* (L.) Lam. | Oleparmunyo | Against common cold, malaria and all other diseases: boil whole plant or make cold-water infusion **Maundu et al.: roots and leaves against malaria** | 57 |
| **Santalaceae** |  |  |  |
| *Osyris lanceolata* Hochst. & Steud. | Olosesai | Remove bark, place on sand to dry, then mix with tea. Makes a very good tea for young and old | 147 |
| **Sapindaceae** |  |  |  |
| *Allophylus abyssinicus* Radlk. | Onchani Orok | No use **Maundu et al.: same** | 71 |
| *Dodonaea angustifolia* L.f. | Enchani Enkashe | When you have migrated from one place to another and you come back to the first place put the branches at the gate. This shows that the cows have come back | 130 |
| *Dovyalis abyssinica* (A. Rich.) Warb. | Olekiku | No use  **Maundu et al.: Roots for soup, gonorrhea, fruits eaten** | 107 |
| **Scrophulariaceae** |  |  |  |
| *Craterostigma plantagineum* Hochst. | Enkorika Emotionyi | When you have pain on sides of body boil and drink 2 cups, also for back pains | 1,48 |
| *Evolvulus alsinoides* (L.) L. | Leleswa Ekopo | 1. Boil and wash yourself when spirit feels bad; 2. Add to fire for sacrifice  **Maundu et al.: No use** | 160 |
| *Striga asiatica* (L.) Kuntze | unknown | No use | 172 |
| **Solanaceae** |  |  |  |
| *Solanum incanum* L. | Entulelei | 1. fruits put in wounds for tiny marks, 2. when you have concussion/swelling make small cuts and crush yellow fruit and rub into small cuts **Maundu et al.: juice of fruit applied to boils and cuts, roots boiled for tooth and throat problems** | 101,134 |
| **Sterculiaceae** |  |  |  |
| *Dombeya burgessiae* Gerrard | Osupukai | 1. Bark used for joining houses; 2. Rub leaves when hands are dirty  **Maundu et al.: strings for construction** | 63,65 |
| **Tiliaceae** |  |  |  |
| *Grewia bicolor* Juss. | Esiteti | 1. Eaten by goats and cows, must NOT be cut for animals; 2. Bark used to join sticks; 3. Toothbrush; 4. Eat ripe (red) fruit | 11,18 |
| *Grewia similis* K. Schum. | Enyalugai | 1. Making sticks; 2. Toothbrush**Maundu et al.: bark for cattle that ate bicarbonate, bark used to wash hair of moran before plaiting, stems for rope in construction, sticks, edible fruit, fodder** | 42 |
| *Grewia tembensis* Fresen. | Esiteti | 1. Eaten by goats and cows, must NOT be cut for animals; 2. Bark used to join sticks; 3. Toothbrush; 4. Eat ripe (red) fruit | 14 |
| *Triumfetta rhomboidea* Jacq. | Olairepirepi | No use but when pods are dry they can enter the eyes and cause damage, fruits stick on clothes | 103 |
| **Family / Scientific name** | **Indigenous name** | **Use and parts used**  **(in bold uses reported by Maundu et al.^14^)** | **Coll.# RBUGGG** |
| **Verbenaceae** |  |  |  |
| *Lippia javanica* Spreng. | Osinoni | Ceremonial - circumcision, put on patients bed afterwards, for good smell, sniff leaves for common cold, eaten by goats | 135A |
| *Priva curtisiae* Kobuski | Elauo Ekop | No use | 10 |
| **Vitaceae** |  |  |  |
| *Cissus rotundifolia* Vahl | Esutai | No use | 20 |
| *Cissus quinquangularis* Chiov. | Osukutut | No use, latex allergenic | 17 |
| ***Indet. (unidentified)*** |  |  |  |
| *indet.* | Enkamererwani | Against common cold, malaria and all other diseases: boil whole plant or make cold-water infusion | 58 |
| *indet.* | Enkiyei | 1. put meat on top of leaves to keep moist for 1-2 days; 2. Toothbrush; 3. Eat sweet berries; 4. Firewood | 69 |
| *indet.* | Enkokii | Bees suck flower | 158 |
| *indet.* | Edalakai | Used by weaver birds to build nests, eaten by cows and buffalos | 54B |
| *indet.* | Ososian | For women in ceremony, old men use it to bless women | 54 |
| *indet.* | unknown | No use | 125 |
